# Application of the International System for Reporting Serous Fluid Cytopathology with Cytohistological Correlation and Risk of Malignancy Assessment

**DOI:** 10.3390/diagnostics11122223

**Published:** 2021-11-28

**Authors:** Alexandros Pergaris, Dimitra Stefanou, Panagiota Keramari, Stylianos Sousouris, Nikolaos Kavantzas, Helen Gogas, Panagiota Mikou

**Affiliations:** 1 Department of Cytopathology, Laiko Hospital, 11527 Athens, Greece; alexperg@yahoo.com (A.P.); giotakeramari@hotmail.com (P.K.); stylianos.s@gmail.com (S.S.); 2 First Department of Pathology, Medical School, National and Kapodistrian University of Athens, 11527 Athens, Greece; nkavantz@med.uoa.gr; 3 First Department of Medicine, School of Medicine, National and Kapodistrian University of Athens, Laiko General Hospital, 11527 Athens, Greece; dimitroulastef@hotmail.com (D.S.); helgogas@gmail.com (H.G.)

**Keywords:** serous effusion, pleural effusion, peritoneal effusion, cancer, cytology, histopathology

## Abstract

The International System for Reporting Serous Fluid Cytopathology (TIS) classifies serous effusions into five categories: non-diagnostic (ND), negative for malignancy (NFM), atypia of unknown significance (AUS), suspicious for malignancy (SFM) and malignant (MAL). The main objectives of this classification comprise the establishment of a universal code of communication between cytopathologists and clinicians and histopathologists, as well as between different laboratories worldwide, paving the way for the setting of clinical management guidelines based on the risk of malignancy assessment for each diagnostic category. We retrieved the total number of pleural and peritoneal effusion cases of our department for the three-year time period between 2018 and 2020, yielding a total of 528 and 500 cases, respectively. We then proceeded to reclassify each specimen according to TIS guidelines and calculate the risk of malignancy (ROM) for each category by searching each patients’ histology records, medical history and clinical follow-up. For pleural effusions, 3 (0.57%) cases were classified as ND, 430 (81.44%) cases as NFM, 15 (2.84%) as AUS, 15 (2.84%) as SFM and 65 (12.31%) as MAL. ROM amounted to 0%, 5.3%, 33.33%, 93.33% and 100% for each category, respectively. As far as peritoneal effusions are concerned, 6 (1.2%) were categorized as ND with ROM estimated at 16.66%, 347 (69.4%) as NFM (ROM = 9%), 13 (2.6%) as AUS (ROM = 38.46%), 12 (2.4%) as SFM (ROM = 83.33%) and 122 (24.4%) as MAL (ROM = 100%). Our results underline the utility of the current classification, both as a means of communication between doctors of different specialties and as general guidelines for the further clinical management of patients.

## 1. Introduction

Serous effusions, both in the pleural and the peritoneal cavities, result from an imbalance between the production and reabsorption of serous fluid [1,2]. Their presence is always considered a pathologic condition, and they reflect a wide range of diseases from benign to malignant [3]. Clinical diagnosis is based on clinical presentation, radiological findings and laboratory testing, including biochemical assays and cytology with or without ancillary techniques and molecular testing [4].

Pleural, peritoneal and pericardial effusions constitute a significant proportion of a cytopathology laboratory workload. Unlike other specimens, such as urinary and bronchial specimens, which represent organ-specific pathology, they are related to pathological conditions of a great variety, including malignancy with origin in many different organs. Material for cytology is easy to obtain, and cytology constitutes a valuable, cost-efficient investigation test.

The cytology of effusions is not restricted to morphology. Microscopic examination is followed by special stains, immunohistochemical stains or flow cytometry, according to the initial morphologic findings [5,6]. In malignant effusions, the classification of the neoplasm as primary (mesothelioma) or secondary (metastasis) is mandatory. Furthermore, the site of origin of the metastatic malignant effusion in patients without or even, occasionally, with a history of malignancy has to be clarified [7,8,9]. Theranostics, a group of molecular assays related to the selection of target therapy for an individual tumor, is an important new field for personalized medicine. Recent literature evidence supports the notion that serous effusions, and even the supernatant fluid after their centrifugation, represent valid material for reliable molecular testing and therapeutic decisions, especially for lung, breast, ovarian and gastrointestinal adenocarcinoma [10,11,12,13,14,15].

Cytology has been reported to have high sensitivity for the diagnosis of malignancy in serous effusions. Nevertheless, a diagnostically gray area, including atypia and suspicious for malignancy cases, exists worldwide, similarly to other areas of cytology. The definition of inadequate and benign samples is also mandatory. The International System for Reporting Serous Effusions (TIS) has been elaborated by an international work group formed by the collaboration of the International Academy of Cytology (IAC) and the American Society of Cytopathology (ASC) in order to clarify these issues and provide a universal reporting scheme following the prototype of similar reporting schemes for cervical specimens and thyroid, salivary gland, breast, urinary and pancreatobiliary systems [16,17,18,19,20,21]. The main objectives comprise the defining of diagnostic criteria, achieving better communication with clinicians and between pathologists in a worldwide spectrum and, finally, setting clinical management guidelines based on risk of malignancy assessment for each diagnostic category [22,23].

In our study, we collected pleural, peritoneal and pericardial effusions that were received in the last three years in our cytopathology laboratory, and we applied the TIS reporting system. Cytohistological correlation was performed in cases with available histology, and the risk of malignancy (ROM) for each category using histology, flow cytometry and clinical follow-up data was assessed.

## 2. Materials and Methods

### 2.1. Data Retrieval

A systematic search of the database of the cytopathology department of Laiko General Hospital was performed in order to retrieve the total number of serous effusion cases between the years 2018 and 2020. This included pleural, peritoneal and pericardial effusions. The standard handling of effusion samples in our laboratory consists of centrifugation and preparation of two conventional smears, one ethanol fixed for Papanicolaou staining and the other air dried for Giemsa staining. Subsequently, residual material is used for liquid-based preparation according to the manufacturer’s instructions (ThinPrep). Liquid-based cytology is performed to provide a better cellular yield, good material for immunostains and to preserve material for molecular studies. Immunocytochemistry (ICC) is mostly performed in ThinPrep slides, and ICC antibodies are all validated prior to use in diagnostic routine, while cellblock preparation is reserved for cases where further workup with molecular studies is needed or in selected cases with a known history that would require a large panel of antibodies. Diagnostic routine in our department is carried out exclusively by specialized cytopathologists. Difficult cases are evaluated by at least two cytopathologists, and a consensus must be reached before making the final diagnosis. The parameters recorded from each cytology report included patients’ age, gender and medical history, as well as each specimen’s volume and ancillary studies, such as immunocytochemistry (ICC), the occasional flow cytometry and final diagnosis.

### 2.2. Previous Reporting Scheme

Our initial reporting system before the application of TIS, based on morphology and ICC, consisted of 4 categories. The first category included inadequate specimens, the second negative for malignancy and the fourth positive for malignancy, either primary or, most usually, secondary. The third category comprised atypical cases, usually accompanied by some comment on the degree of atypia and the likelihood of malignancy. The cases in the atypical category were practically redistributed to TIS3, atypia of undetermined significance, and TPS4, suspicious for malignancy. Reevaluation of the cytology reports was conducted by two specialized cytopathologists (P.K. and P.M.), who were aware of the initial diagnosis. The slides were reevaluated, and the initial cytology report of each case also proved particularly useful. Most of the time, the description in each report classified roughly, more or less, each case into one of the five TIS categories.

### 2.3. The International System for Reporting Serous Effusions (TIS)

After reevaluation, each cytology report was classified into one of the following categories, according to the TIS:

- ND: Non-diagnostic specimen.
- NFM: Specimen negative for malignancy.
- AUS: Presence of atypical cells that, however, lack evidence of malignancy, with the atypical characteristics often attributed to inflammatory changes. Of note, specimens classified as AUS tend to lean closer to the benign end of the spectrum.
- SFM: Presence of cells with atypia not enough for a diagnosis of malignancy, but such diagnosis strongly indicates malignancy.
- MAL: Specimens containing clearly malignant cells.

### 2.4. Cytohistological Correlation and Further Analysis

The database of the histopathology department of our hospital was investigated for histological diagnosis matching our cases, either previous, synchronous or following the cytology report. Clinical information and radiologic findings were also noted. Clinical files were also searched in order to collect information on the patient disease course. All the aforementioned data were inserted into an Excel spreadsheet for further processing.

Risk of malignancy (ROM) assessment and evaluation of diagnostic performance were both based on a combination of histology when available and clinical follow-up in the rest of the cases. For accurate parameter evaluation, ND, NFM and AUS were considered negative and SFM and MAL positive results.

## 3. Results

The total number of pleural and peritoneal effusion specimens in the three-year period between 2018 and 2020 amounted to 528 and 500 cases, respectively. There were also three pericardial effusions. Their distribution to the TIS categories is presented in Table 1.

### 3.1. Pleural Effusions

**Figure 1:**
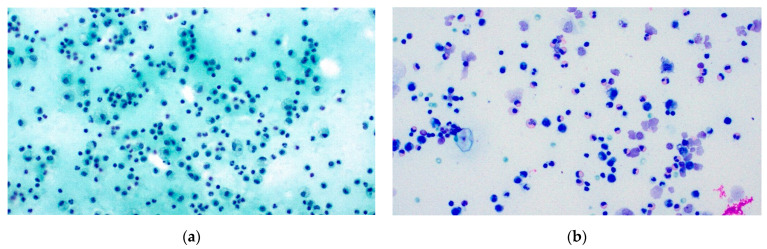
Pleural effusion NFM (TIS2). (**a**) Reactive effusion, Pap stain ×200, and (**b**) eosinophilic effusion, Giemsa stain ×200.

**Figure 2:**
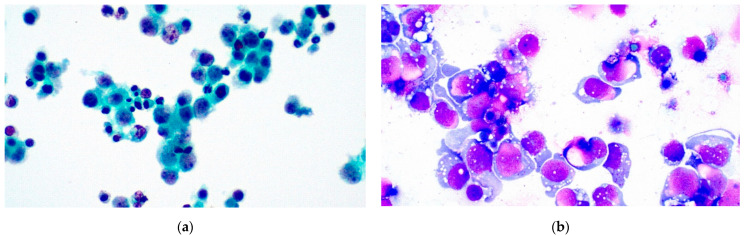
Pleural effusion MAL (TIS5) diffuse large B-cell lymphoma (DLBCL). (**a**) Pap stain ×200 and (**b**) Giemsa stain ×400.

Pleural effusion cases consisted of 286 male and 242 female patients (male to female ratio = 1.18), with age ranging from 11 to 95 years old and median age equal to 68.92 years. Mean specimen volume amounted to 173.75 mL (ranging from 0.1 to 1600 mL). Of 528 cases in total, 3 were classified as ND (0.57%), 430 as NFM (81.44%), 15 as AUS (2.84%), 15 as SFM (2.84%), and 65 (12.31%) were positive for malignancy. ICC was conducted in 46 of the cases. Data are presented in Table 2, and examples of benign and malignant pleural effusions are shown in Figure 1 and Figure 2.

Sixteen of the sixty-five cases diagnosed as malignant were accompanied by a histological report. Meanwhile, the remaining 49 cases were accompanied by a patients’ medical history, including previous histology, consistent with our diagnoses. The most common site of malignancy origin was the breast (12), followed by the lung (10) and lymphoma (8).

Risk of malignancy assessment was based on available histology and clinical records, including radiologic findings and previous histology. Fifty-five NFM pleural effusion cases were lost to follow-up. Thus, risk of malignancy was assessed for the remaining 375 NFM cases. ROM was 0%, 5.3%, 33.33%, 93.33% and 100% for ND, NFM, AUS, SFM and MAL, respectively.

Diagnostic performance in our laboratory was also evaluated for cases with data availability, considering ND, NFM and AUS as negative and SFM and MAL as positive results. Sensitivity was 75.9%, specificity 99.7%, PPV 98.75% and NPV 93.6%.

### 3.2. Peritoneal Effusions

**Figure 3:**
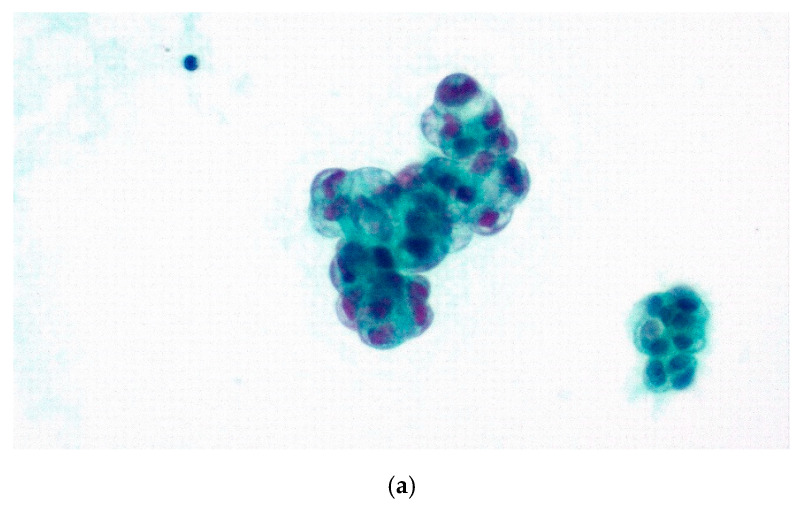

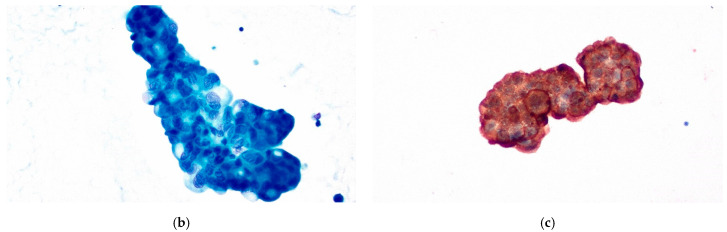
Peritoneal effusion, MAL (TIS5), pancreatic adenocarcinoma. (**a**) Pap stain ×400, (**b**) Giemsa stain ×400 and (**c**) CEA immunostain ×400.

As far as peritoneal effusion cases are concerned, 246 cases were males and 254 females (male to female ratio = 0.97%), with age ranging from 16 to 93 years old, median age amounting to 67.6 years and mean specimen volume to 234.72 mL (ranging from 0.2 to 2400 mL). As shown in Table 1, 6 cases were categorized as ND (1.2%), 347 as NFM (69.4%), 13 as AUS (2.6%), 12 as SFM (2.4%) and 122 (24.4%) as MAL, with a total of 69 cases accompanied by IHC studies. Data are presented in Table 3, and an example of a malignant case is shown in Figure 3.

Our search in the database of the pathology department retrieved 76 concomitant histology reports, while the remaining 46 cases were also accompanied by a medical history, including previous histology, compatible with the type of malignancy reported in our cytology reports. The most common site of malignancy origin was the ovaries (39), followed by the stomach (19) and the breast (15). Table 4 presents the most common site of malignancy for pleural and peritoneal effusions.

Forty-nine NFM peritoneal effusion cases were lost to follow-up. Thus, the risk of malignancy was assessed for the remaining 298 NFM cases. The ROM was calculated as 16.66% for ND, 9% for NFM, 38.46% for AUS, 83.33% for SFM and 100% for MAL specimens. The ROM for each of the five categories of pleural and peritoneal effusion cases is presented on Table 5.

Diagnostic performance evaluation resulted in 80% sensitivity, 99.3% specificity, 98.5% PPV and 89.6% NPV.

### 3.3. Pericardial Effusions

There were only three cases of pericardial effusions, one of which was reactive, related to trauma, and two malignant, with lung cancer origin (Table 1). Due to the small number of cases, no further analysis was performed.

## 4. Discussion

In this study, the application of the International System for Reporting Serous Effusions (TIS) was evaluated based on serous effusion cytology over a period of three years in a tertiary general hospital. Serous effusions represent a significant proportion of the cytopathology laboratory annual workload. Clinicians utilize cytology to diagnose or exclude malignancy and to obtain additional information on the cell consistency of the sample, which is critical for the etiologic definition of the effusion and therapeutic management.

During this period, 528 pleural, 500 peritoneal and 3 pericardial effusions were processed in the laboratory. Reevaluation with distribution to the TIS categories showed that pleural effusions were 0.57% ND, 81.44% NFM, 2.84% AUS, 2.84% SFM and 12.31% MAL. Peritoneal effusions were 1.22% ND, 70.39% NFM, 2.64% AUS, 2.43% SFM and 24.75% MAL. Pericardial effusions only included three cases, and their distribution was 33.33% (one case) NFM and 66.67% (two cases) MAL. Our results are compatible with those of other studies [24,25,26,27,28,29,30,31,32], some of which include only pleural effusions [25,26,27,30], while others include pleural and peritoneal effusions [24,28,31,32], and one includes only pericardial effusions [29]. In regard to pleural effusions, the rate of malignancy reported from oncologic centers is significantly higher [31], while a similar rate to ours is reported from other institutions [26], probably related to the “general” nature of our institution.

The most common malignancy in pleural effusions was secondary to breast cancer, followed by lung cancer and lymphoma. A similar order of secondary malignancy origin has been reported by other studies [25,26,30,31]. A well-developed hematologic clinic in our hospital accounts for the relatively high incidence of lymphomas and leukemias in our series, which, surprisingly, was much higher before the COVID-19 era. The COVID-19 pandemic has seriously affected the total annual number of serous effusion cases. There were no primary malignancies (mesotheliomas) in our series. These cases, along with most lung cancers, are concentrated in two specialized pulmonary hospitals in Athens. The gastrointestinal system (51 cases) and the gynecological system, mostly the ovaries (36 cases), are the most common sites of origin of adenocarcinomas spreading in the peritoneal cavity in our series, which is similar to another study [31]. The gynecological system, especially the ovaries, is the most common site of origin of malignant ascites in women. Of note, the incidence of each tumor organ of origin partly depends on the distribution of patients among the various hospitals in our city. Many breast and gynecological cancer cases are forwarded to our institution for workup and treatment, elevating the incidence of relative malignancies in our study. Both malignant pericardial effusions represented the spread of lung cancer, and the benign effusion was related to trauma. Lung cancer has been reported as the main cause of malignant pericardial effusion in a study with a large series of pericardial effusions [29].

In our hospital, pleural and peritoneal biopsies are confined to cases with strong radiologic evidence and clinical suspicion of malignancy and negative or indeterminate effusion cytology. In these cases, repeat paracentesis samples may lead to diagnosis, avoiding more interventional investigation. Thus, most of the histological correlation in our series is based on biopsies of the primary tumor. Especially in cases with no previous history, morphologic evaluation in conjunction with radiologic evidence and other clinical data leads to a selection of immunocytochemical markers, allowing the effusion cytological diagnosis to clarify, where possible, the origin of the tumor, and subsequent biopsy or surgery is scheduled. Our policy is to utilize a small panel of antibodies not only for cost-efficiency issues but also for a better evaluation and clarity of the results. In cases with a previous history of malignancy, morphologic and immunocytochemical compatibility with the known primary is considered a final diagnosis. In patients with suspected hematologic malignancy, either with or without previous history, the cytology diagnosis is assisted by flow cytometry [33].

In our series, the risk of malignancy, ROM, was evaluated for ND, NFM, AUS, SFM and MAL as 0%, 5.3%, 33.33%, 93.33% and 100%, respectively, for pleural effusions. As far as peritoneal effusions are concerned, the ROM for the aforementioned categories was evaluated as 16.67%, 9%, 38.46%, 83.33% and 100%, respectively. A comprehensive review by Farahani and Baloch [24] reported mean ROM for all types of serous effusions of 17.4%, 20.7%, 65.9%, 81.8% and 98.9% for the abovementioned TIS categories. Other studies have reported mean ROM ranging between 40% and 87.5% for ND, 20.16% and 51.61% for NFM, 39% and 88.23% for AUS, 64% and 87.5% for SFM and 100% for MAL [25,26,28,31]. Our results are more compatible with the ROM reported by Kundu et al. and Xu et al., namely 20% and 26.7% ND, 12% and 16.7% NFM, 50% and 62.3% AUS, 77.8% and 94.4% SFM and, finally, 100% for MAL [30,32]. It seems that the absence of false positives with ROM of 100% in the malignant (TIS5) category is a universal finding. The high PPV (100%) of serous effusion cytology is indisputable. Discrepancies in the reports of different studies are inevitable, as the study material and clinical management throughout different institutions are variable. However, we consider ROM assessment more valuable as significant information to the clinicians in an individual institution.

The diagnostic performance in our laboratory was evaluated. For pleural effusions, sensitivity was 75.9%, specificity 99.7%, PPV 98.75% and NPV 93.6%. Meanwhile, for peritoneal effusions, the corresponding values were 80%, 99.3%, 98.5% and 89.6%. The accuracy parameter values are in line with those reported in other studies [25,26,27,28,29,30,31,32]. Notably, oncologic centers report lower sensitivity [25,31]. In general hospitals such as ours, most of the negative cases are true negatives, as the clinics handle patients with a wide range of nonneoplastic conditions. The presence of false negatives in our series is due to either extremely small or extremely large sample volume or tumors with spread to the serosal membranes without shedding tumor cells in the fluids extracted. Large sample volume can pose a diagnostic challenge in cases where malignant cells are present in small numbers, as they can prove difficult to detect. In such cases, in the setting of strong clinical suspicion for malignancy, the process of centrifugation and slide preparation is repeated many times over, and multiple slides are prepared. Similarly, specimens with minimal volumes of a few milliliters can also pose a diagnostic challenge, especially in the presence of overtly malignant cells, where there is no material available for further ICC studies to identify the organ of origin. The ideal specimen volume, as well as the minimum and maximum volumes, is still a matter of discussion between cytopathologists. A consensus has not yet been reached on what constitutes the minimum or maximum specimen volume where, beyond the end of its spectrum, proper diagnosis is compromised. The overall diagnostic performance of serous effusion cytology with the application of the TIS is very good, justifying the utilization of the new reporting system and cytology in the workup of patients presenting with serous effusions.

We classified a total of 58 cases as false negative, as concomitant histopathology reports or clinical information revealed the presence of malignancy. Nonetheless, we noticed that, in many cases, the disease was not advanced, and the effusions could be attributed to other pathologic conditions. For example, a peritoneal effusion of a female patient that was later diagnosed with stage 1 endometrial carcinoma would fall under the classification of false negative. It is obvious, however, that a restricted disease of such a low stage could not have shed malignant cells in the peritoneal cavity, and the presence of ascites should be attributed to different pathological processes. This is part of an extensive discussion between cytopathologists that remains to be conducted so that consensus can be reached regarding what constitutes a false negative diagnosis as far as pleural, peritoneal and pericardial effusions are concerned. Other reasons for a false negative result include extremely small or large sample volumes and tumors with spread to the serosal membranes without shedding tumor cells in the fluids extracted. False positive cases (one pleural and two peritoneal cases) were due to overdiagnosis of reactive effusions with highly atypical mesothelial cells and no residual material for ICC in one case and inconclusive ICC in the other two cases.

Our experience with application of the TIS in our material was generally positive. The criteria used for the definition of each category are clear and easy to apply as reported elsewhere [26]. A better definition of the optimal specimen volume as to the upper limit is essential, as extremely large volumes of fluid are difficult to process, and malignant cells can be missed. Additionally, during the execution of this study, clinicians appreciated the new system, especially after the assessment of the ROM for each category.

The first limitation of this study is the rarity of pleural and peritoneal biopsies for cytohistological correlation. Consequently, surgical biopsies of the primary tumor were mostly used. Of course, a series analysis including pleural and peritoneal effusions that were all followed by pleural and peritoneal biopsies, respectively, could possibly overestimate ROM, as most of the time, such biopsies are conducted when strong clinical suspicion of malignancy is present. Another limitation is related to the nature of a general hospital, providing inevitable discrepancies to the reports from oncological hospitals.

## 5. Conclusions

The International System for Reporting Serous Effusions (TIS) is easy to use and exhibits high accuracy values for serous effusion cytology. It is also helpful in the communication with the clinicians, as the criteria of adequacy, optimal volume definition and ROM assessment for each category contribute to the improvement of the clinical service.

## Figures and Tables

**Table 1:** Number and distribution of pleural, peritoneal and pericardial effusions per TIS category.

	ND	NFM	AUS	SFM	MAL	Total
**Pleural**	3 [0.57%]	430 [81.44%]	15 [2.84%]	15 [2.84%]	65 [12.31%]	528 [100%]
**Peritoneal**	6 [1.2%]	347 [69.4%]	13 [2.6%]	12 [2.4%]	122 [24.4%]	500 [100%]
**Pericardial**	0 [0%]	1 [33.33%]	0 [0%]	0 [0%]	2 [66.67%]	3 [100%]

**Table 2:** Number of pleural effusion cases and gender, age, volume, ICC studies and histology report for each TIS category.

Diagnostic Category	Gender		Age (Years)	Volume (mL)	ICC	Histology Reports
ND (*n* = 3)	M: 3	F: 0	Min: 11	Min: 1	0	Total: 2
			Max: 83	Max: 10		Synchronous: 2
			Ave: 48.67	Ave: 5.33		Previous: 0
NFM (*n* = 430)	M: 249	F: 181	Min: 11	Min: 0.1	26	Total: 202
			Max: 95	Max: 1600		Synchronous: 89
			Ave: 67.37	Ave: 182		Previous: 13
AUS (*n* = 15)	M: 2	F: 5	Min: 60	Min: 0.5	1	Total: 3
			Max: 83	Max: 50		Synchronous: 3
			Ave: 75	Ave: 19.38		Previous: 0
SFM (*n* = 15)	M: 11	F: 4	Min: 55	Min: 3	10	Total: 14
			Max: 72	Max: 700		Synchronous: 14
			Ave: 66	Ave: 125.57		Previous: 0
MAL (*n* = 65)	M: 21	F: 44	Min: 59	Min: 0.5	9	Total: 16
			Max: 92	Max: 1400		Synchronous: 13
			Ave: 74	Ave: 206.5		Previous: 3
Total (*n* = 528)	M: 286	F: 242	Min: 11	Min: 0.1	46	Total: 239
			Max: 95	Max: 1600		Synchronous: 215
			Ave: 68.92	Ave: 173.75		Previous: 16

**Table 3:** Number of peritoneal effusion cases and gender, age, volume, ICC studies and histology report for each TIS category.

Diagnostic Category	Gender		Age (Years)	Volume (mL)	ICC	Histology Reports
ND (*n* = 6)	M: 3	F: 3	Min: 58	Min: 3	0	Total: 0
			Max: 88	Max: 20		Synchronous: 0
			Ave: 70.75	Ave: 8.7		Previous: 0
NFM (*n* = 347)	M: 187	F: 160	Min: 16	Min: 0.2	28	Total: 184
			Max: 89	Max: 2400		Synchronous: 32
			Ave: 66.47	Ave: 230.44		Previous: 152
AUS (*n* = 13)	M: 8	F: 5	Min: 42	Min: 5	3	Total: 8
			Max: 85	Max: 500		Synchronous: 0
			Ave: 64.6	Ave: 133.2		Previous: 8
SFM (*n* = 12)	M: 6	F: 6	Min: 55	Min: 3	2	Total: 12
			Max: 87	Max: 100		Synchronous: 4
			Ave: 70	Ave: 36.5		Previous: 8
MAL (*n* = 122)	M: 42	F: 80	Min: 35	Min: 1	36	Total: 76
			Max: 93	Max: 2000		Synchronous: 3
			Ave: 70.38	Ave: 245.2		Previous: 73
Total (*n* = 500)	M: 246	F: 254	Min: 16	Min: 0.2	69	Total: 268
			Max: 93	Max: 2400		Synchronous: 39
			Ave: 67.6	Ave: 234.72		Previous: 226

**Table 4:** Cancer site of origin in malignant pleural and peritoneal effusion cases.

Pleural Effusion Cases (65)	
Breast	12
Lung	10
Lymphoma	8
Pancreas	7
Ovary	7
Colon	6
Leukemia	5
Angiosarcoma	2
Urothelial carcinoma	2
Melanoma	2
Multiple myeloma	2
Esophagus	1
Stomach	1
**Peritoneal Effusion Cases (122)**	
Ovary	36
Stomach	19
Breast	15
Colon	14
Pancreas	14
Urothelial carcinoma	8
Melanoma	5
Bile duct	3
Gallbladder	2
Lung	2
Lymphoma	2
Leukemia	1
Renal cell carcinoma	1

**Table 5:** Risk of malignancy (ROM) for the TIS categories in pleural and peritoneal effusions.

	ND	NFM	AUS	SFM	MAL
**PLEURAL**	(0/3) 0%	(20/375) 5.3%	(5/15) 33.33%	(14/15) 93.33%	(65/65) 100%
**PERITONEAL**	(1/6) 16.66%	(27/298) 9%	(5/13) 38.46%	(10/12) 83.33%	(122/122) 100%

## Data Availability

The data presented in this study are available on request from the corresponding author.
